# A Newly Discovered Genetic Disorder Associated With Life-Threatening Aortic Disease in a 6-Year-Old Boy

**DOI:** 10.1177/2324709620909234

**Published:** 2020-02-26

**Authors:** Mohanad Hamandi, Madison L. Bolin, Joy Fan, Allison T. Lanfear, Seth K. Woolbert, Ronald D. Baxter, J. Michael DiMaio, William T. Brinkman

**Affiliations:** 1 Baylor Scott and White—The Heart Hospital, Plano, TX, USA

**Keywords:** ARIH1, aortic aneurysm, aortic dissection

## Abstract

Aortic aneurysms in children are rare and when present are usually caused by a connective tissue disorder. In this article, we present a case of multiple aortic aneurysms in an adolescent with a novel finding of a gene variation that is associated with aortic disease.

## Introduction

Aortic disease in children is rare and primarily caused by connective tissue disorders.^1^ Each aortic pathology is characterized by a genotype variation and corresponding clinical expression. The most common cause is Marfan syndrome, which affects 0.02% to 0.03% of the population.^1^ Among athletes who presented with aortic dilation, 40% were found to have Marfan syndrome.^2^ Loeys-Dietz syndrome is another prevalent connective tissue disorder with vascular implications, causing aortic root dilation in 95% of those diagnosed and 50% in the remaining portion of the aorta.^1,3^ Vascular Ehlers-Danlos syndrome is also a connective tissue disorder, affecting up to 1 in 100 000 people.^1^ The most common cause of death in patients with vascular Ehlers-Danlos syndrome is arterial dissection.^1^ These syndromes are known to cause aortic aneurysms and dissections.^1^ Further pathologies are being investigated to determine other causes of aortic diseases in children. We present a novel finding of a gene variation that is associated with aortic disease in a young patient.

## Case Summary

A 6-year-old male was referred for evaluation of an incidentally discovered ascending aortic aneurysm in 2005. The patient did not manifest signs or symptoms of connective tissue disorder, autoimmune disease, or constitutional illness on examination, and both of his parents are healthy. Thoracic aortic imaging of his father, brother, and paternal half-brother revealed no pathological findings. Computed tomography angiography confirmed a 4.7-cm fusiform aneurysm extending from the aortic root to the ascending aorta, for which the cardiothoracic team successfully performed aortic root and ascending aortic replacement with a mechanical valve conduit (Bentall procedure). Six years later, the patient returned with atypical chest pain and was found to have an acute type B dissection extending distally that required an emergent open replacement of his descending thoracic aorta. He was managed medically for the remainder of his descending aneurysm but subsequently required an open thoracoabdominal aortic repair with Dacron graft in 2013 for continued dilatation and increasing back pain. The patient was closely followed postoperatively, and in 2014, an aortic arch aneurysm with right brachiocephalic artery involvement was found. He then underwent total arch replacement with debranching. Genetic testing for *COL3A1* (Ehlers-Danlos), *TGFβR1, TGFβR2* (Loeys-Dietz), *ACTA2* (familial thoracic aortic aneurysm and dissection), and *SMAD3* (Loeys-Dietz and familial thoracic aortic aneurysm and dissection) was negative. The patient was referred for further investigation with whole exome sequencing and gene matching at a specialized institute. The investigators previously studied *Drosophila* (fruit fly) and noted that the mutant *ari-1* gene had pleiotropic phenotypes and sought to investigate whether mutations in the corresponding human variant (*ARIH1*) induce disease. Tan et al report that mutations of *ari-1* in *Drosophila* are responsible for the clustering of nuclei in striatal muscle as compared with the wild-type larvae.^4^ Following this discovery, the patient was 1 of 3 who was found to have a rare variant of *ARIH1* gene. *ARIH1* variants expressed in *Drosophila* showed failure in rescuing nuclear positioning of *ari-1* mutants and did not survive for more than a few days, concluding that variants result in loss of function of ARIH1 protein.^4^ Further studies on the nuclei of aortic smooth muscle cells of patient samples versus control samples showed morphological irregularity, likely induced by functional loss of *ARIH1*.^4^ Mechanosensing, the ability of a cell to respond to cellular environment changes, is dependent on functional contractile units of the cell, which include adhesions to the nuclear membrane.^4^ The nuclear envelope is disrupted in *ARIH1* variants causing complications in mechanosensing and may be accountable for the weakening of the aortic wall muscles and inducing aneurysms.^4^

## Discussion

Aortic disease is mainly caused by Marfan syndrome, Loeys-Dietz syndrome, vascular Ehlers-Danlos syndrome, and familial thoracic aortic aneurysm and dissection.^1,4,5^ The most common connective tissue disorder is Marfan syndrome, characterized by dislocation of the ocular lens and skeletal deformities due to an inherited mutation in fibrillin-1.^1^ This mutation leads to loss of elastic properties and stiffness of the aortic wall leading to dilation and dissection.^1^ Delmo et al report the presence of aneurysms of the aortic root and ascending aorta in 30.6% of patients in a study of cardiovascular interventions in children with Marfan syndrome.^6^ To confirm the presence of Marfan syndrome, a scoring system considering physical features, *FBN1* mutation, and cardiovascular abnormalities is used.^7^ The patient did not have family history or the clinical features associated with Marfan syndrome. Loeys-Dietz syndrome is an autosomal-dominant connective tissue disorder with a mutation in the genes encoding for TFG-β receptors 1 and 2.^1^ The clinical features expressed are similar to Marfan syndrome through loss of elastin and increased collagen synthesis and is characterized by ascending aortic aneurysms and dissection.^1^ The patient tested negative for variation in *SMAD3, TFGR1*, and *TFGR2*, in which mutation is characteristic of Loeys-Dietz Syndrome.^3^ A third genetic disorder causing aortic disease is vascular Ehlers-Danlos syndrome; mutation of the *COL3A1* gene is causal of tears of the aorta and its major branches in which dissection is common.^1^ There are typical clinical expressions, including facial appearance effects, thin skin, arterial rupture, and gravid uterus or intestine.^1^ Shalhub et al reported that 17.6% of patients with Ehlers-Danlos syndrome had type B aortic dissection.^8^ The patient tested negative for a mutation in *COL3A1* as well. Furthermore, genetic testing for familial thoracic aortic aneurysm and dissection was also performed, yielding negative results. Table 1 summarizes the most common syndromes with their corresponding clinical features and genotype variation. These negative genetic results, in conjunction with no family history of aortic disease, led to further investigation at a specialized genetic institute. It was found that a rare variant of *ARIH1* in the patient’s exome may have caused this aortic disease. We believe that this finding revealed a novel cause of aortic pathology that was not previously known as a potential cause for aortic aneurysms and dissections.

**Table 1.** Indications of Genetic Disorders Related to Aortic Disease.

Disease	Primary Location of Aortic Disease	Clinical Indications	Genetic Testing
Marfan syndrome^1,5^	Aortic root	Annuloaortic ectasia	*FBN1*
		Ectopic lens	
		Long bone overgrowth	
Loeys-Dietz syndrome^1,3^	Ascending aorta	Hypertelorism	*TGFβ2*, *TGFβ3*, *TGFβR1*, *TGFβR2*, *SMAD2*, *SMAD3*
		Bifid uvula	
		Cleft palate	
		Arterial tortuosity	
Ehlers-Danlos syndrome^1,5^	Aorta and major branches	Emaciated face	*COL3A1*, *COL5A1*, *COL5A2*
		Thin skin	
		Arterial rupture	
		Gravid uterus or intetine	
		Easy bruising	
Familial thoracic aortic aneurysm and dissection^5,9^	Thoracic aorta	Fixed dilated pupils	*ACTA2*, *TFGβR2*, *MYH11*
		Hypotonic bladder	
		Intestinal hypoperistalsis	
		Pulmonary hypertension	

## Conclusion

**Figure 1.**
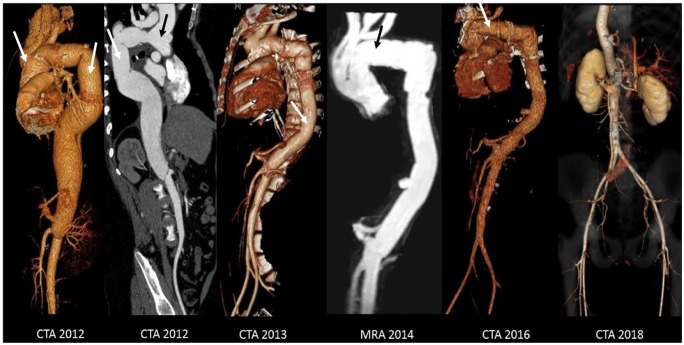
Computed tomography angiography and magnetic resonance angiography serial follow-ups. Various procedures were performed before or after shown scans: Bentall procedure (2005) and descending thoracic replacement (2011); thoracoabdominal replacement (2013); and aortic arch replacement (2014). New surgical repairs are denoted by arrows.

This patient’s mutation in his *ARIH1* gene may be the cause of his life-threatening aortic disease that has been successfully managed at our institution.^4^ The patient is currently alive and attending college with minimal limitations related only to his need for oral anticoagulation. Since the long-term prognosis of his remaining arterial vasculature is unknown, we plan to continue a close follow-up schedule with computed tomography angiography of the thorax, abdomen, and pelvic vasculature. Serial follow-up computed tomography angiography and magnetic resonance angiography imaging are regularly performed (Figure 1).
